# Chemical crosslinking analysis of β-dystroglycan in dystrophin-deficient skeletal muscle

**DOI:** 10.12688/hrbopenres.12846.2

**Published:** 2018-09-17

**Authors:** Sandra Murphy, Margit Zweyer, Rustam R. Mundegar, Dieter Swandulla, Kay Ohlendieck

**Affiliations:** 1 Department of Biology, Maynooth University, National University of Ireland, Maynooth, Maynooth, Co. Kildare, Ireland; 2 Institute of Physiology II, University of Bonn, Bonn, D‑53115, Germany

**Keywords:** Bis[sulfosuccinimidyl]suberate, Cavin-1, Chemical crosslinking, Duchenne muscular dystrophy, Dysferlin, Dystroglycan, Dystrophin, Dystrophinopathy, Myoferlin

## Abstract

**Background**: In Duchenne muscular dystrophy, primary abnormalities in the membrane cytoskeletal protein dystrophin trigger the loss of sarcolemmal linkage between the extracellular matrix component laminin-211 and the intracellular cortical actin membrane cytoskeleton. The disintegration of the dystrophin-associated glycoprotein complex renders the plasma membrane of contractile fibres more susceptible to micro-rupturing, which is associated with abnormal calcium handling and impaired cellular signalling in dystrophinopathy.

**Methods**: The oligomerisation pattern of β-dystroglycan, an integral membrane protein belonging to the core dystrophin complex, was studied using immunoprecipitation and chemical crosslinking analysis. A homo-bifunctional and non-cleavable agent with water-soluble and amine-reactive properties was employed to study protein oligomerisation in normal versus dystrophin-deficient skeletal muscles. Crosslinker-induced protein oligomerisation was determined by a combination of gel-shift analysis and immunoblotting.

**Results**: Although proteomics was successfully applied for the identification of dystroglycan as a key component of the dystrophin-associated glycoprotein complex in the muscle membrane fraction, mass spectrometric analysis did not efficiently recognize this relatively low-abundance protein after immunoprecipitation or chemical crosslinking. As an alternative approach, comparative immunoblotting was used to evaluate the effects of chemical crosslinking. Antibody decoration of the crosslinked microsomal protein fraction from wild type versus the *mdx-4cv* mouse model of dystrophinopathy revealed oligomers that contain β-dystroglycan. The protein exhibited a comparable reduction in gel electrophoretic mobility in both normal and dystrophic samples. The membrane repair proteins dysferlin and myoferlin, which are essential components of fibre regeneration, as well as the caveolae-associated protein cavin-1, were also shown to exist in high-molecular mass complexes.

**Conclusions**: The muscular dystrophy-related reduction in the concentration of β-dystroglycan, which forms in conjunction with its extracellular binding partner α-dystroglycan a critical plasmalemmal receptor for laminin-211, does not appear to alter its oligomeric status. Thus, independent of direct interactions with dystrophin, this sarcolemmal glycoprotein appears to exist in a supramolecular assembly in muscle.

## Introduction

Duchenne muscular dystrophy, the most frequently inherited neuromuscular disorder of early childhood, is caused by primary abnormalities in the *Dmd* gene ^1^. The full-length protein product of the *Dmd* gene, the Dp427-M isoform of dystrophin ^2^, functions as a membrane cytoskeletal protein and molecular anchor for a variety of associated glycoproteins and cytosolic components in normal muscle ^3– 5^. In dystrophinopathy, the almost complete loss of dystrophin triggers the collapse of the dystrophin-associated glycoprotein complex at the sarcolemmal membrane system ^6^. A pathobiochemical hallmark of X-linked muscular dystrophy is the significant reduction in dystroglycans, sarcoglycans, sarcospan, dystrobrevins and syntrophins ^7^. This renders dystrophic muscle fibres more susceptible to micro-rupturing of the plasmalemma, which in turn causes complex secondary disturbances of excitation-contraction coupling, calcium homeostasis, proteostasis and cellular signalling ^8^. The proteomic profiling of muscular dystrophy has established complex alterations in protein families involved in the maintenance of the cytoskeletal network, the extracellular matrix, ion handling, the excitation-contraction-relaxation cycle, the cellular stress response and bioenergetic pathways ^9– 11^.

Following the initial discovery of the core dystrophin-glycoprotein complex ^12^, detailed biochemical, cell biological and proteomic investigations focusing on the wider dystrophin complexome have established a variety of additional protein species that exist in close proximity to the actin-dystrophin-dystroglycan-laminin axis in skeletal muscle fibres. This includes the neuronal isoform nNOS of nitric oxide synthase, biglycan, vimentin, tubulin, synemin, desmin, cytokeratin and the desmoglein/desmoplakin-complex ^13– 19^. In dystrophin-deficient fibres, many of the closely associated elements of the dystrophin complex are reduced in abundance, while cytoskeletal proteins such as vimentin and desmin are up-regulated to rescue the impaired structural integrity of dystrophic cells ^20^. Crucial membrane repair proteins, such as myoferlin and dysferlin, were also shown to increase in their density in the sarcolemma of dystrophic muscles ^21^.

Since the disintegration of the supramolecular dystrophin-glycoprotein complex initiates the multifaceted pathogenesis of dystrophinopathy, which is characterized by highly progressive fibre degeneration, sterile inflammation, fatty tissue replacement and reactive myofibrosis ^22^, it was of interest to study the oligomeric status of a key component of this complex, the integral glycoprotein β-dystroglycan ^23^, in normal versus dystrophic muscles. The recent proteomic analysis of muscular dystrophy using a combination of chemical crosslinking, gel-shift analysis and mass spectrometry has confirmed the exclusive presence of dystrophin in high-molecular-mass complexes in normal muscle membranes ^24^. However, other components of the core dystrophin complex could not be identified by mass spectrometry, which is probably due to their relatively low abundance, small size and/or hydrophobicity. In dystrophin-deficient preparations, oligomerized protein species were identified as sarcolemmal components, cytoskeletal proteins, structural elements of the extracellular matrix and mitochondrial proteins ^24^. These altered protein-protein interaction patterns are probably related to cellular repair mechanisms, re-structuring of the stabilizing surface matrix of contractile fibres and metabolic adaptations to counteract the dystrophic phenotype.

In this report, we have evaluated the bioanalytical potential of immunoprecipitation and chemical crosslinking analysis for studying the oligomerisation pattern of β-dystroglycan, in wild type versus dystrophic muscle specimens from the *mdx-4cv* mouse model of Duchenne muscular dystrophy. For the chemical crosslinking analysis, the 11.4-Å crosslinker bis[sulfosuccinimidyl]suberate (BS ^3^) was used, which is homo-bifunctional, water-soluble, non-cleavable and amine-reactive making this agent highly suitable for analyses of protein interactions under physiological conditions ^25^. A combination of one-dimensional gel-shift analysis and immunoblotting established the presence of oligomers that contain β-dystroglycan in both wild type and dystrophic muscle membranes. Hence, the drastic reduction of the dystrophin-associated glycoprotein β-dystroglycan in muscular dystrophy does not seem to affect its oligomeric status in the sarcolemma.

## Methods

### Materials

General analytical grade reagents and materials for chemical crosslinking, immunoprecipitation, gel electrophoresis, mass spectrometry and immunoblotting were supplied by Sigma Chemical Company (Dorset, UK), Bio-Rad Laboratories (Hemel-Hempstead, Hertfordshire, UK), GE Healthcare (Little Chalfont, Buckinghamshire, UK) and National Diagnostics (Atlanta, GA, USA). The homo-bifunctional and amine-reactive crosslinker bis[sulfosuccinimidyl]suberate (BS ^3^) of 11.4-Å spacer arm length (catalogue number 21580) and C18 spin columns (catalogue number 89870) were purchased from Thermo Fisher Scientific (Dublin, Ireland). Chemiluminescence substrate (catalogue number 11500694001) and protease inhibitor cocktails (catalogue number 11836153001) were obtained from Roche Diagnostics (Mannheim, Germany). Biobasic C18 Picofrit columns (catalogue number 72105-254630) were from Dionex (Sunnyvale, CA, USA). Proteolytic digestion for the generation of peptides prior to mass spectrometric analysis was carried out with sequencing grade modified trypsin (catalogue number V5111) from Promega (Madison, WI, USA). Whatman nitrocellulose transfer membranes (catalogue number 88018) came from Invitrogen (Carlsbad, CA, USA). Primary antibodies were purchased from the following companies: Abcam, Cambridge, UK (polyclonal antibody ab43125 to β-dystroglycan, RRID: AB_955822, used at a dilution of 1:250; rabbit polyclonal antibody ab190264 to myoferlin (product code ab190264), used at a dilution of 1:250; monoclonal antibody ab124684 to dysferlin; RRID: AB_10976241, used at a dilution of 1:100), Novus Biologicals, Littleton, CO, USA (polyclonal antibody NBP1-80220 to cavin 1/PTRF (polymerase and transcript release factor); RRID: AB_11019160) and Santa Cruz Biotechnology, Santa Cruz, CA, USA (monoclonal antibody sc-33701 to β-dystroglycan; RRID: AB_627294). Merck-Millipore (MA, USA) provided peroxidase-conjugated secondary antibodies [catalogue numbers AQ132P (anti-rabbit) and 12-349 (anti-mouse)].

### Isolation of the microsomal fraction from skeletal muscle

**Figure 1.**
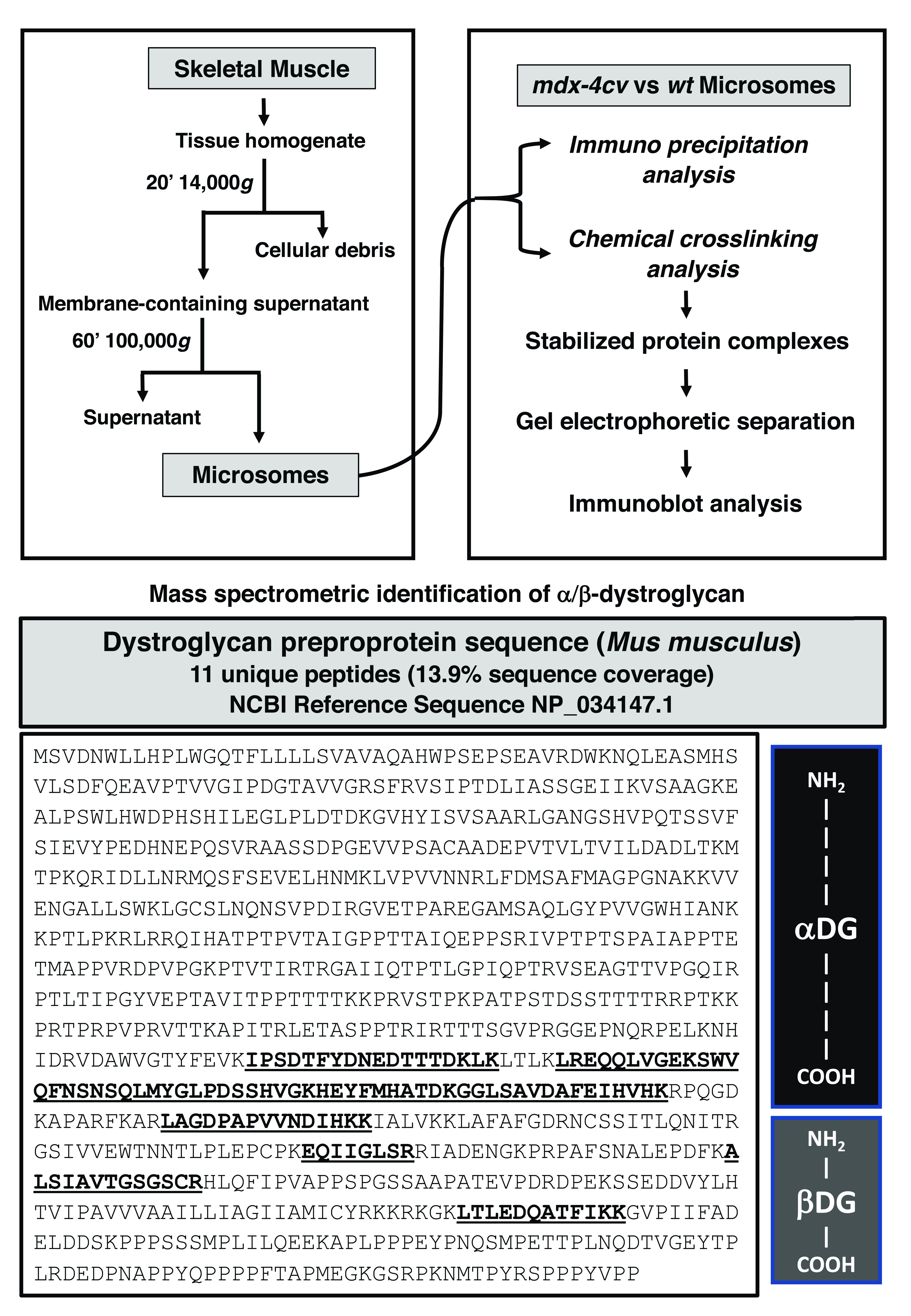
Bioanalytical workflow to identify β-dystroglycan and determine its oligomeric status in muscle membranes. Shown is an overview of the differential centrifugation scheme to isolate the crude microsomal fraction from wild type versus dystrophic *mdx-4cv* skeletal muscle homogenates. To evaluate potential protein interactions, both immunoprecipitation and chemical crosslinking in combination with gel-shift and immunoblot analysis were employed. The presence of dystroglycans in microsomes was established by mass spectrometry, which identified a considerable number of unique peptide sequences that are characteristic for both the α-subunit and the β-subunit of the dystroglycan subcomplex. The lower panel shows the peptide sequence of the pre-pro-protein of 893 amino acids including the signalling peptide and both dystroglycan subunits, which approximate portions of the entire protein sequence are marked on the right. The sequences of unique peptides are in bold and underlined.

A crude microsomal membrane fraction was isolated from wild type and dystrophic *mdx-4cv* muscle samples, which were obtained from the Animal Facility of the University of Bonn ^26^. Transportation of quick-frozen muscle specimens to Maynooth University was carried out on dry ice in accordance with the Department of Agriculture animal by-product register number 2016/16 to the Department of Biology, Maynooth University. As outlined in the flow chart of Figure 1, combined skeletal muscles of the hind leg from 5-month old dystrophic *mdx-4cv* mice (n=4) and age-matched C57Bl/6 mice (n=4) were finely chopped and then homogenised on ice with the help of a hand-held IKA T10 Basic Homogeniser (IKA Labortechnik, Staufen, Germany). Homogenisation was performed in 10 volumes of buffer (20mM sodium pyrophosphate, 20mM sodium phosphate, 1mM MgCl _2_, 0.303M sucrose, 0.5mM EDTA, pH 7.0) that was supplemented with a protease inhibitor cocktail from Roche Diagnostics (Mannheim, Germany). The suspension was incubated for 2h at 4°C and centrifuged at 14,000× *g* for 20 min at 4°C with a model 5417 R centrifuge from Eppendorf (Hamburg, Germany) to remove cellular debris. The membrane-containing supernatant was transferred to 4.9ml Optiseal ultracentrifugation tubes and centrifuged at 100,000× *g* for 1h at 4°C using an Optima L-100 XP ultracentrifuge from Beckman Coulter Incorporation (Fullerton, CA, USA). The resulting pellets with the crude microsomal membrane fraction were re-suspended in an appropriate volume of homogenisation buffer and then stored at -20°C until usage for mass spectrometric analysis, immunoprecipitation or chemical crosslinking. The protein concentration of individual samples was determined by the method of Bradford ^27^.

### Immunoprecipitation analysis of β-dystroglycan in normal skeletal muscle

The evaluation of the immunoprecipitation technique ^28^ as a potential bioanalytical approach for the identification of dystroglycan-associated proteins ^17^ was carried out with crude microsomes that had been isolated from C57Bl/6 mice. Two distinct antibodies to β-dystroglycan were used, i.e. ab43125 from Abcam (Cambridge, UK) and sc-33701 from Santa Cruz Biotechnology (Santa Cruz, CA, USA). Magnetic beads coupled to Protein A (for antibody ab43125) or Protein G (for antibody sc-33701) were used for the pre-clearance of 1mg of membrane protein using an incubation step for 30 min at 4°C with end-over-end rotation. The supernatant was isolated and incubated with 5µg antibody overnight at 4°C with gentle end-over-end rotation. The following day, the suspension of protein and antibody was added to 25µl of magnetic beads and incubated for 1h at room temperature with end-over-end rotation. The supernatant was removed using a magnetic rack, and the beads were washed five times, thrice with Tris-buffered saline with 0.05% Tween-20 and twice with distilled water. Proteins were subsequently eluted in one of two ways. Proteins were eluted first with 2M urea, 50mM Tris-HCl, pH 7.5 and 5µg/ml trypsin and incubated at 27°C for 30 min with gentle shaking. This supernatant was collected, and the magnetic beads were then washed twice with 2M urea, 50mM Tris-HCl, pH 7.5, 1mM dithiothreitol. The supernatant from this step was combined with the supernatant from the first elution. The samples were left to digest overnight at room temperature. The following day, 20µl of 5mg/ml iodoacetamide was added and incubated at room temperature in the dark for 30 min. 2% trifluoroacetic acid (TFA) in 20% acetonitrile (ACN) (3:1 (v/v) dilution) was then added and peptides were purified using C18 spin columns before mass spectrometry. Alternatively, after the five washes of the magnetic beads, proteins were eluted with Laemmli-type buffer ^29^ and incubated at 97°C for 7 min. These protein samples were digested by the FASP (filter aided sample preparation) method ^30^ and purified by C18 spin columns. In both cases, peptides were re-suspended in loading buffer (2% ACN, 0.05% TFA in LC-MS grade water), and were analysed by a Q-Exactive mass spectrometer over a 65 min gradient. Raw files obtained from mass spectrometry were analysed by Proteome Discoverer 1.4 and filtered for high confidence peptides.

### Comparative chemical crosslinking analysis using bis(sulfosuccinimidyl)suberate

The water-soluble cross-linker bis(sulfosuccinimidyl)suberate (BS ^3^) was dissolved at a concentration of 1mg/ml in 50mM citrate buffer, pH 5.0 ^31^. Microsomal samples were diluted to a concentration of 2 mg protein/ml with 50mM HEPES, pH 8.0. A concentration range of 10, 25, 50, 75, 100 and 150μg BS ^3^ per mg protein was used to evaluate the crosslinking protocol employed in this study ^24^. For the comparative crosslinking and immunoblot analysis of β-dystroglycan, cavin-1, myoferlin and dysferlin oligomerisation, membrane samples were incubated for 30 min at 25°C with 0 versus 10µg BS ^3^ per mg protein. The addition of 50μl 1M ammonium acetate per ml reaction mixture was used to quench chemical crosslinking and then an equal volume of reducing sample buffer was added to the samples. Following a heating step for 10 min at 50°C, crosslinked proteins were separated by one-dimensional gel electrophoresis alongside their non-crosslinked counterparts ^24^.

### Label-free liquid chromatography mass spectrometric analysis

Protein digestion and mass spectrometric analysis of peptide populations was carried out by an optimized method ^26^. Following pre-treatment of the microsomal fraction with the Ready Prep 2D clean up kit (catalogue number 163-2130) from Bio-Rad Laboratories (Hemel-Hempstead, Hertfordshire, UK), pellets obtained were re-suspended in label-free solubilisation buffer (6 M urea, 2 M thiourea, 10 mM Tris, pH 8.0 in LC-MS grade water) and protein suspension volumes were equalised with label-free solubilisation buffer. Samples were reduced with dithiotreitol and alkylated with iodoacetamide, followed by the proteolytic digestion using a combination of the enzymes Lys-C and trypsin, as previously described in detail ^32^. The digestion step was stopped by acidification with 2% TFA in 20% ACN (3:1 (v/v) dilution). Peptide suspensions were purified using Pierce C18 Spin Columns from Thermo Fisher Scientific (Dublin, Ireland) and the resulting peptide samples were dried through vacuum centrifugation and suspended in loading buffer consisting of 2% ACN and 0.05% TFA in LC-MS grade water. An Ultimate 3000 NanoLC system (Dionex Corporation, Sunnyvale, CA, USA) coupled to a Q-Exactive mass spectrometer (Thermo Fisher Scientific), situated in the Proteomics Suite of Maynooth University, was used for the label-free liquid chromatography mass spectrometric (LC-MS/MS) identification of dystroglycan. Peptide mixtures were loaded by an autosampler onto a C18 trap column (C18 PepMap, 300 µm id × 5 mm, 5 µm particle size, 100-Å pore size; Thermo Fisher Scientific). The trap column was switched on-line with an analytical Biobasic C18 Picofrit column (C18 PepMap, 75 µm id × 50 cm, 2 µm particle size, 100-Å pore size; Dionex). Peptides generated from muscle microsomal proteins were eluted using the following binary gradient solvent A [2% (v/v) ACN and 0.1% (v/v) formic acid in LC-MS grade water] and 0–90% solvent B [80% (v/v) ACN and 0.1% (v/v) formic acid in LC-MS grade water]: 0–2% solvent B for 10.5 min, 2–40% solvent B for 110 min, 40–90% solvent B for 2.5 min, 90% solvent B for 9 min and 2% solvent B for 43 min. Peptides generated from immunoprecipitation were eluted over the same binary gradient, with 0–90% solvent B over a 65 min gradient. The column flow rate was set to 0.25 µl/min. Data were acquired with Xcalibur software version 2.0.7 (Thermo Fisher Scientific). The mass spectrometer was operated in positive mode and data-dependent mode and was externally calibrated. Survey MS scans were conducted in the Q-Exactive mass spectrometer in the 300–1700 m/z range with a resolution of 140,000 (m/z 200) and lock mass set to 445.12003. CID (collision-induced dissociation) fragmentation was carried out with the fifteen most intense ions per scan and at 17,500 resolution. Within 30s, a dynamic exclusion window was applied. An isolation window of 2 m/z and one microscan were used to collect suitable tandem mass spectra. Mass spectrometry files were analysed by Proteome Discoverer 1.4 (Thermo Fisher Scientific) software with Sequest HT as the search engine and the UniProtKB-SwissProt sequence database. The following search parameters were used for protein identification: (i) peptide mass tolerance set to 10 ppm, (ii) MS/MS mass tolerance set to 0.02 Da, (iii) up to two missed cleavages, (iv) carbamidomethylation set as a fixed modification, (v) methionine oxidation set as a variable modification and vi) peptide probability set to high confidence.

### Immunoblot analysis of chemically crosslinked muscle proteins

A comparative immunoblotting approach was employed to evaluate the potential shift in electrophoretic mobility of muscle protein species following chemical crosslinking ^33– 35^. For immunoblotting, microsomes were suspended in 2x standard Laemmli-type buffer for one-dimensional sodium dodecyl sulfate polyacrylamide gel electrophoresis (SDS-PAGE) ^29^, heated for 10 min at 50°C and then loaded onto hand-cast 10% SDS-PAGE gels. Following gel electrophoretic separation, proteins were transferred to nitrocellulose membranes, blocked in a fat-free milk protein solution (2.5% milk powder in 10% phosphate-buffered saline) and incubated 1.5 h in an appropriately diluted solution with primary antibody ^36^. Immuno-decorated membranes were carefully washed and incubated overnight with peroxidase-conjugated secondary antibodies. Antibody-labelled protein bands were visualized with the enhanced chemiluminescence technique ^37^.

## Results

In this report, we outline the findings from a study on the oligomerisation pattern of β-dystroglycan, an integral sarcolemma protein that is greatly reduced in X-linked muscular dystrophy. Chemical crosslinking in combination with gel-shift analysis and immunoblotting was successfully applied to determine changes in the electrophoretic mobility pattern of this core member of the dystrophin-associated glycoprotein complex.

### Mass spectrometric identification of β-dystroglycan in the microsomal fraction from skeletal muscle

Mass spectrometry-based proteomics was used to identify dystroglycan in the muscle membrane fraction. As a key component of the dystrophin-associated glycoprotein complex, the products of the *DAG1* gene (that encodes dystroglycan with the sequence ID NP_034147.1) form a sub-complex of the core dystrophin network, which links the extracellular matrix protein laminin-211 of the basal lamina and the subsarcolemmal actin cytoskeleton in skeletal muscle. The encoded pre-pro-protein of 895 amino acids (aa), which includes the signalling peptide and both dystroglycan subunits ^38^, undergoes extensive O- and N-glycosylation, as well as proteolytic processing to generate extracellular α-dystroglycan and the integral sarcolemma protein β-dystroglycan in rabbit muscle ^36^. The equivalent pre-pro-protein from mice shows a high degree of sequence homology and consists of 893 aa. The mass spectrometric survey of mouse skeletal muscle microsomes identified unique peptide sequences that cover both the α-subunit (aa 28–651) and the β-subunit (aa 652–893) of the dystroglycan subcomplex ( Figure 1). However, mass spectrometric analysis did not efficiently recognize this relatively low-abundance protein after immunoprecipitation or chemical crosslinking. As an alternative approach, comparative immunoblotting was used to evaluate the effects of chemical crosslinking.

### Immunoprecipitation analysis of β-dystroglycan

Prior to chemical crosslinking analysis, we attempted to study potential interactions between β-dystroglycan and closely associated muscle proteins using one of the most frequently employed methods for the investigation of protein-protein interactions, i.e. co-immunoprecipitation. However, this approach resulted in a large number of precipitated protein species that appeared to lack a high degree of specificity, so this bioanalytical approach was therefore not further pursued. Antibody-based precipitation analysis led to the identification of 228 proteins with ≥ 2 unique peptides using the two-step urea-based elution method with the polyclonal antibody ab43125, and 211 proteins with ≥ 2 unique peptides with the monoclonal antibody sc-33701. Elution with Laemmli-buffer resulted in the identification of 273 proteins with ≥ 2 unique peptides with the ab43125 antibody and 245 proteins with ≥ 2 unique peptides with the sc-33701 antibody. Interesting co-immunoprecipitated proteins, which were previously shown to interact directly or indirectly with dystrophin ^5^, included α-sarcoglycan, β-sarcoglycan, δ-sarcoglycan, γ-sarcoglycan, desmin, synemin, dysferlin, tubulin, cytokeratin, vimentin, tetraspanin and desmoplakin. However, dystroglycan (Q62165) was recognized by only 2 peptides, which equates to 2% coverage of the *DAG1* sequence. This was deemed not to be efficient enough for a reliable oligomerization analysis of this dystrophin-associated glycoprotein.

### Chemical crosslinking analysis of β-dystroglycan

An established non-cleavable and homo-bifunctional agent with amine-reactive and water-soluble properties, bis[sulfosuccinimidyl]suberate (BS ^3^), was used to evaluate patterns of protein clustering in the microsomal membrane fraction from normal versus dystrophin-deficient skeletal muscles. Artefacts of random crosslinking are known challenges in chemical crosslinking experiments, although it has been suggested that only those proteins that are non-randomly closely associated will become crosslinked with sufficient frequency to be detected amongst the background of all other proteins. To minimize any potential artefacts, conditions regarding buffer composition, temperature, pH-value and length of incubation time, parameters which have been previously optimised by our laboratory for muscle membrane proteins ^24, 31, 33– 35^, were carefully controlled. In particular, the protein-crosslinker conjugation reaction was carried out under experimental conditions, which are relatively close to physiological conditions and low levels of crosslinker were used (10µg BS ^3^ per mg protein) ^24^.

**Figure 2.**
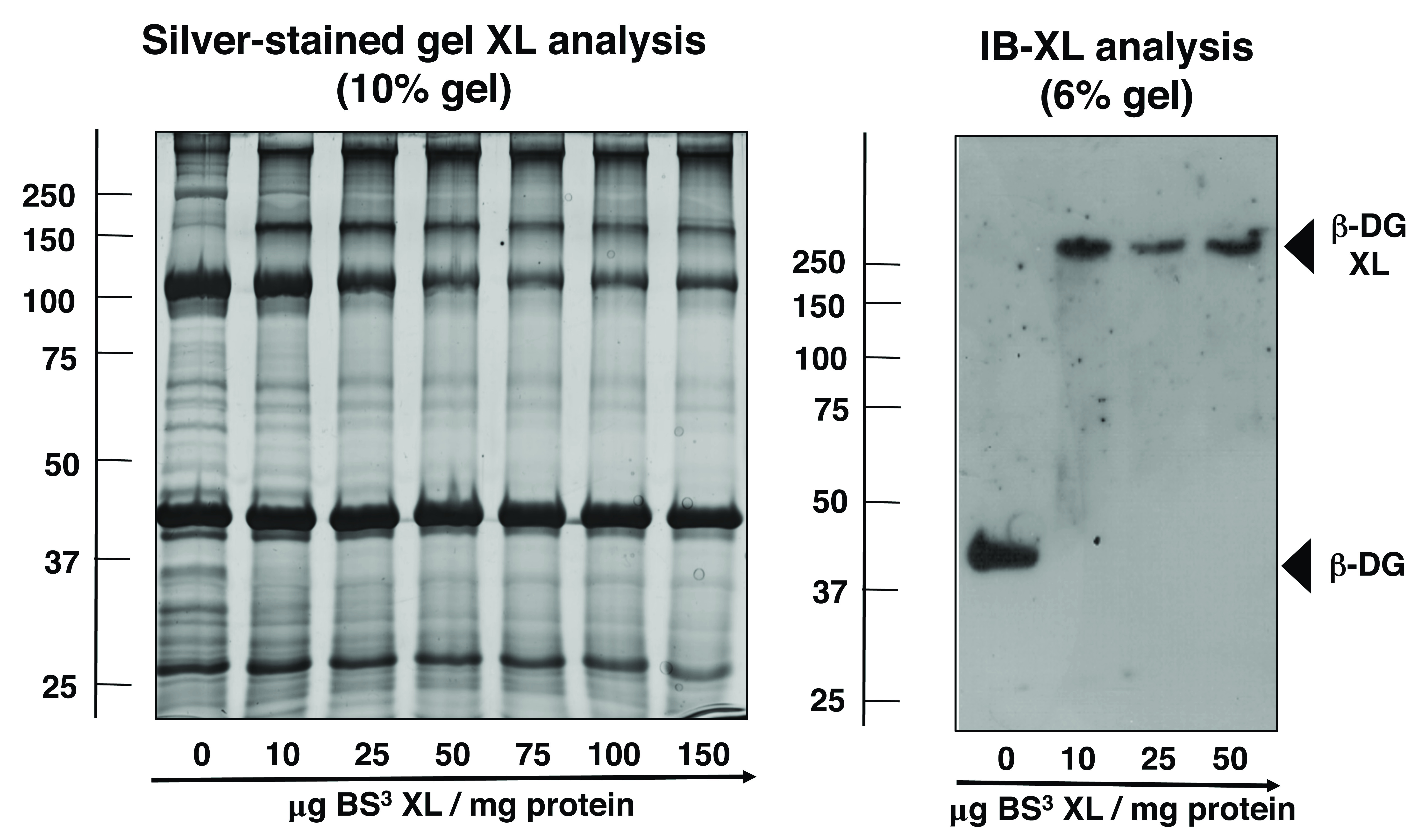
Chemical crosslinking, gel shift and immunoblot analysis of β-dystroglycan from skeletal muscle membranes. Shown is a silver-stained 10% SDS-PAGE gel of normal mouse muscle microsomes that were incubated with 0, 10, 25, 50, 75, 100 and 150 μg of the homo-bifunctional, non-cleavable, water-soluble and amine-reactive 11,4-Å cross-linker bis(sulfosuccinimidyl)suberate (BS³) per mg membrane protein, as well as a corresponding immunoblot that has been transferred from a 6% gel and labelled with an antibody to the dystrophin-associated glycoprotein β-dystroglycan. Molecular mass standards (in kDa) are indicated on the left of panels. The position of monomeric versus crosslinker-stabilized oligomeric forms of β-dystroglycan (DG) are marked by arrowheads.

The degree of chemical crosslinker-induced protein oligomerisation was determined by a combination of one-dimensional gel-shift analysis and immunoblotting. Building on the recently established and optimized protocol for the comparative chemical crosslinking analysis of muscular dystrophy ^24^, antibody decoration of β-dystroglycan was used to study the oligomeric status of this sarcolemmal glycoprotein. As illustrated in the silver-stained 10% SDS-PAGE gel in Figure 2, increasing amounts of BS ^3^ had a considerable influence on the protein banding pattern with the appearance of a distinct band above the 150 kDa gel zone. Immunoblotting of crosslinked complexes was carried out with a 6% gel system in order to capture the potential appearance of proteins with a shift to very high molecular mass. The immunoblot in Figure 2 demonstrated a clear shift to reduced electrophoretic mobility of the monomeric 43 kDa band of β-dystroglycan to a region of above 250 kDa following incubation with BS ^3^.

**Figure 3.**
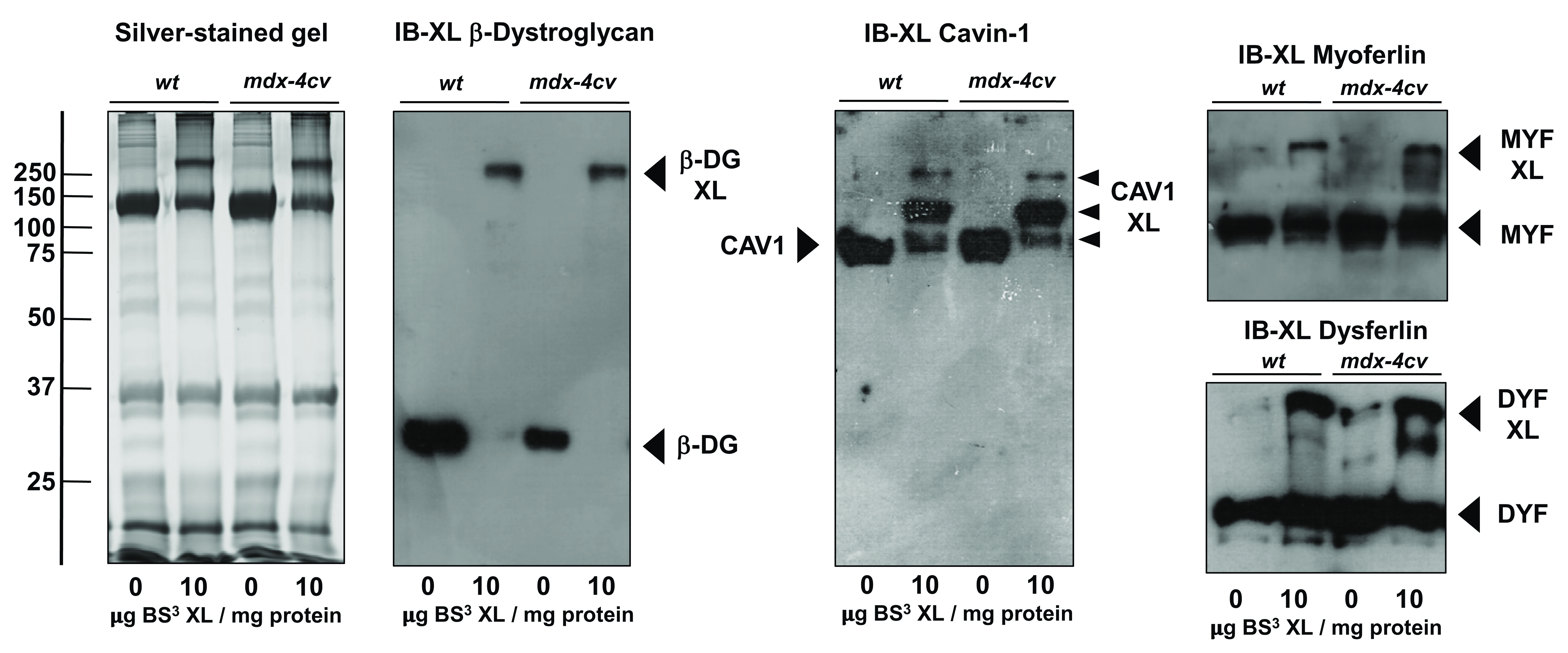
Comparative chemical crosslinking analysis of β-dystroglycan, cavin-1, myoferlin and dysferlin from normal versus dystrophic muscle. Shown is a silver-stained SDS-PAGE gel of muscle microsomes from wild type and dystrophic *mdx-4cv* skeletal muscles and corresponding immunoblots that were labelled with antibodies to β-dystroglycan, cavin-1, myoferlin and dysferlin. Lanes 1 to 4 represent microsomal samples from wild type ( *wt*) and *mdx-4cv* muscles that were incubated with 0 versus 10 μg of the cross-linker bis(sulfosuccinimidyl)suberate (BS ^3^) per mg protein, respectively. Molecular mass standards (in kDa) are indicated on the left of the gel panel. The position of monomeric versus crosslinker (XL) -stabilized oligomeric forms of β-dystroglycan (DG), cavin-1 (CAV1) myoferlin (MYF) and dysferlin (DYF) are marked by arrowheads.

**Figure 4.**
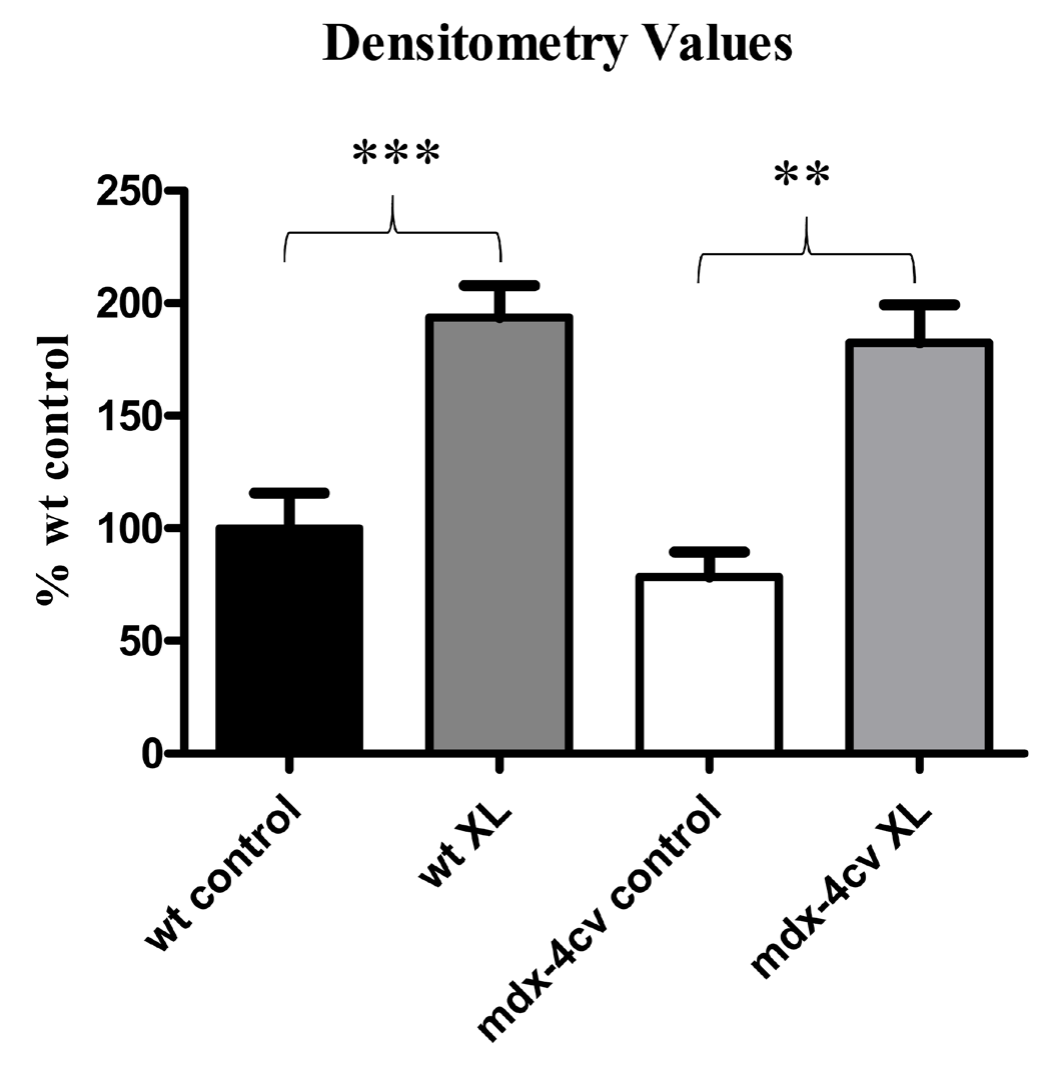
Densitometric scanning of chemical crosslinking and gel-shift analysis of normal versus dystrophic muscle. Shown are densitometric values, which reflect the extent of protein staining in wild type control, wild type crosslinked, *mdx-4cv* control and *mdx-4cv* crosslinked samples. The comparative densitometric evaluation was focused on protein species above 150 kDa. Statistical analysis was performed with a Student’s *t*-test (mean value ±SEM; n=4; **p≤0.01; ***p≤0.001).

The comparative analysis of the crosslinked microsomal protein fraction from wild type versus the *mdx-4cv* mouse model of dystrophinopathy, using an optimized ratio of 10μg BS ^3^ per mg microsomal protein, revealed oligomers that contain β-dystroglycan ( Figure 3). The densitometric analysis of the silver-stained gel illustrates statistically significant increases in densitometry values corresponding to increased protein staining in high-molecular mass regions of the gel above 150 kDa for both wild type and dystrophic crosslinked samples ( Figure 4). Hence, chemical crosslinking resulted in the stabilisation of protein complexes and reduced electrophoretic mobility of protein species, resulting in an increased number of proteins remaining at high molecular mass regions following gel electrophoretic separation. Interestingly this sarcolemma-spanning key component of the core dystrophin complex exhibited a comparable reduction in gel electrophoretic mobility in both normal and dystrophic samples.

Immunoblotting with antibodies to cavin-1 identified this caveolae-associated protein in both control and crosslinked samples from both wild type and *mdx-4cv* muscle preparations ( Figure 3). In control lanes, a cavin-1 band was identified at approximately 80 kDa, which is higher than the previously observed electrophoresed protein band of the apparent monomer at 43 kDa, so this band might present a dimeric structure. The immuno-decoration at a higher molecular mass may also be due to post-translational modifications, abnormal electrophoretic mobility patterns and/or incomplete protein reduction during sample preparation for gel electrophoretic separation. In both wild type and *mdx-4cv* crosslinked preparations, cavin-1 was detected at lower amounts at approximately 80 kDa than in control lanes, and 3 additional bands were evident at approximately 100, 150 and 260 kDa ( Figure 3). This indicates that cavin-1 may be involved in a number of protein interactions giving rise to these 3 distinct bands in crosslinked muscle samples. The fainter upper cavin-1 band of approximately 260 kDa overlaps with the position of crosslinked β-dystroglycan, which suggests that a certain proportion of cavin-1 molecules may exist in a protein complex with β-dystroglycan.

The membrane repair proteins dysferlin and myoferlin, which play a central role in fibre regeneration and counteract the dystrophic phenotype, were also shown to exist in high-molecular mass complexes in normal and dystrophic muscle ( Figure 3). However, in contrast to β-dystroglycan, both sarcolemmal repair proteins only showed a portion of their monomeric protein species to shift to a higher molecular mass range. This limited reduction in electrophoretic mobility of myoferlin and dysferlin in both wild type and dystrophic skeletal muscle, upon the addition of the BS ^3^ chemical cross-linker, suggests that only a sub-population of these proteins form tight protein complexes. The presence of additional bands representing these proteins in *mdx-4cv* muscle indicates that in dystrophic muscle these proteins probably form additional protein complexes in a dynamic fashion, and that some of these protein assemblies are not present in wild type skeletal muscle.

## Discussion

Dynamic interaction patterns between proteins play a key role in the maintenance of complex biomembrane structures. The disintegration of these essential protein-protein interaction patterns during disease processes may have detrimental consequences for cellular signalling events and membrane stability. In X-linked muscular dystrophy, the breakdown of the dystrophin-dystroglycan core and its associated glycoprotein complex represents such a pathobiochemical event and illustrates the importance of supramolecular protein assemblies for normal skeletal muscle function ^4^. In this report, we have focused on the biochemical analysis of the integral component of the crucial trans-sarcolemmal axis of skeletal muscle fibres, consisting of laminin-211, α-dystroglycan, β-dystroglycan, dystrophin isoform Dp427-M and cortical actin ^5^. The glycoprotein β-dystroglycan was shown to exist in a high-molecular-mass complex in microsomal membranes that can be stabilized by a suitable chemical crosslinking agent that exhibits 11.4-Å spacer arm length, and this oligomeric status appears to be maintained even in the absence of the molecular anchor protein dystrophin. One of the most interesting technical aspects of this study is the demonstration that the oligomerisation of low-abundance proteins can be evaluated by relatively simple immuno-decoration methods.

To study the three-dimensional structure of proteins and protein-protein interaction patterns in health and disease, a variety of sophisticated biochemical methods are routinely used, which include nuclear magnetic resonance spectroscopy, X-ray crystallography, Förster resonance energy transfer, cryo-electron microscopy, yeast two-hybrid screening, co-immunoprecipitation, gel filtration and native two-dimensional gel electrophoresis. More recently, new applications of advanced mass spectrometry have also been employed to study protein structure and protein complex formation ^39^. Within this relatively new field of structural mass spectrometry, the combination of chemical crosslinking and proteomics has shown great promise to study both transient protein assemblies and extremely large protein complexes ^40^. However, low copy number proteins can sometimes not be properly studied by peptide or protein mass spectrometry due to a low signal-to-noise ratio. Since antibodies are usually highly specific for a distinct antigenic determinant, they can often overcome these bioanalytical limitations of protein detection ^31^. In the case of immunoblotting of chemically crosslinked complexes, only those types of antibodies can be successfully employed that recognize both the monomeric and oligomeric forms of their respective antigen ^33– 35^. This has been the case with the antibody to β-dystroglycan used in this report, which could thus be utilized as an alternative bioanalytical tool for the visualization of crosslinked complexes.

The drastic reduction in dystrophin-associated proteins, including the integral component β-dystroglycan, appears to be the primary triggering mechanism that renders the surface membrane of dystrophic muscle fibres more susceptible to contraction-induced micro-rupturing ^6^. A leaky sarcolemma membrane with an impaired physiological integrity, in combination with natural repair mechanisms, was shown to cause abnormal plasmalemmal calcium fluxes ^8^. A decreased buffering capacity for calcium ions in both the cytosol and the lumen of the sarcoplasmic reticulum probably enhances this disturbance of ion homeostasis in dystrophinopathy ^41– 43^. Chronically elevated calcium levels in the sarcosol result in the activation of proteolytic processes that are thought to be at the core of fibre degeneration in X-linked muscular dystrophy ^44^. In addition, skeletal muscle necrosis is associated with complex secondary pathophysiological changes, including a robust inflammatory response, the substitution of dystrophic fibres with fatty cells and progressive tissue scarring due to reactive myofibrosis ^45^.

 The findings from the comparative chemical crosslinking analysis presented here confirm the significant reduction in β-dystroglycan in the *mdx-4cv* mouse model of Duchenne muscular dystrophy ^3, 46^. Prior to the biochemical stabilization of the dystroglycan-containing complex, immunoblotting clearly demonstrated a lower concentration of the 43 kDa monomer. However, the gel-shift and immunoblot analysis of crosslinked β-dystroglycan showed a comparable reduction in electrophoretic mobility of its complexed form in normal versus dystrophic muscle, which is a surprising result. Thus, despite the lack of full-length dystrophin and a considerable reduction in α-dystroglycan, the sarcoglycan sub-complex, sarcospan, dystrobrevins and syntrophins ^6^, the integral membrane linker of the core dystrophin complex appears to exist in a high-molecular-mass assembly. This suggests that, despite its greatly decreased concentration, the oligomeric status of β-dystroglycan is preserved in dystrophin-deficient muscle fibres. These protein interactions might be maintained by the remaining members of the dystrophin-associated glycoprotein complex.

Interestingly, the sarcolemma-associated protein cavin-1 was previously shown to interact with β-dystroglycan ^47^. The adapter protein cavin and the structural caveolin proteins mediate the formation and organization of caveolae. These bulb-like membrane pits represent small invaginations in the sarcolemma and form specialized lipid micro-domains. Sarcolemmal caveolae mediate a variety of processes, including cell signalling, endocytosis, lipid storage and mechano-protection ^48^. The shift of both β-dystroglycan and cavin-1 to approximately 260 kDa is in agreement with the study by Johnson and co-workers ^47^. However, in contrast to the previous observation that binding of cavin-1 to β-dystroglycan is lost in *mdx* mice suggesting that this caveolae component is reduced in the absence of dystrophin, in our study cavin-1 was detected at 260 kDa in both wild type and *mdx-4cv* muscle microsomes. Therefore, further analysis would be required in order to determine whether the cavin-1 containing 260 kDa band also incorporates β-dystroglycan or whether this assembly represents a different cavin-1 complex.

Myoferlin and dysferlin appear to also exist, at least partially, in large protein aggregates in both normal and dystrophic fibres. Both sarcolemmal proteins are crucial players of regulatory processes in skeletal muscle tissue, such as calcium homeostasis and the formation of transverse tubules ^49^. In muscular dystrophy, interactions between dysferlin, myoferlin, actin and various annexins are involved in sarcolemmal resealing via vesicular patching, fusion and restoration processes that counteract membrane disintegration due to the lack of the cytoskeletal stabilizer dystrophin ^50^. The observed oligmerisation of myoferlin and dysferlin might relate to the initiation of the membrane repair process and the maintenance of sarcolemmal integrity. The more diverse protein complex formation of dysferlin and myoferlin in *mdx-4cv* membranes, as compared to wild type muscle, may be a compensatory mechanism of dystrophic skeletal muscle to mitigate some of the secondary pathobiochemical effects, which arise as a result of the loss of dystrophin. Indeed, our research group has recently used chemical crosslinking combined with mass spectrometry to investigate alterations in the oligomerisation of high-molecular-mass protein complexes in dystrophic skeletal muscle ^24^. This analysis identified 23 proteins in dystrophic microsomal fractions, which displayed an electrophoretic mobility shift to very high molecular mass, of which 15 proteins were only found in dystrophic microsomes and 8 were detected in both wild type and *mdx-4cv* microsomes. One of these 15 proteins was identified as myoferlin ^24^, which may interact with caveolin, actin and myosin-9 in high-molecular-mass complexes, and facilitate sarcolemmal membrane repair. Since membrane repair is a well-described compensatory mechanism in dystrophic muscle ^50^, which is partially mediated by myoferlin and dysferlin, heightened interactions of these proteins with other repair proteins in muscular dystrophy may account for the additional protein complexes seen in dystrophic muscle.

In recent years a number of groups have utilised chemical crosslinking as a means of studying protein interactions in the context of the dystrophin-associated glycoprotein complex. Royuela and colleagues ^51^ have used the chemical N, N’-p-phenylenedimaleimide (pPDM) on enriched β-dystroglycan preparations from skeletal and cardiac muscle and brain from *Xenopus laevis*. Immunoblotting revealed that the 38 kDa β-dystroglycan preparation from skeletal muscle gave rise to a doublet band of 125 kDa, which was suggested to correspond to interactions between β-dystroglycan and α-dystrobrevin ^51^. Although smaller in size than the crosslinked band of approximately 260 kDa established in this report, it is possible that the 260 kDa band may similarly correspond to a complex of β-dystroglycan with syntrophins and dystrobrevins, although this would need to be confirmed by immunoblotting. The pPDM agent used in the previous study ^51^ is based on a chemical interaction between two different cysteine groups or between one cysteine group and a lysine group. This reaction pattern differs from the BS ^3^ XL agent used in this study, which links two primary amine groups together. The use of different cross-linking agents is also useful for determining spatial constraints given that they differ in their spacer arm length. For example, chemical crosslinkers with extremely short spacer arm lengths, such as N-succinimidyl-iodoacetate, can only stabilise the interactions between proteins that exist in very close proximity to another.

Our laboratory has previously used the 1,5-difluoro-2,4-dinitrobenzene crosslinker of shorter arm length than the BS ^3^ agent utilized in this study to examine the cardiac dystroglycan complex ^52^. A crosslinker-stabilised protein complex of approximately 200 kDa was detected, which probably represents tightly associated α/β-dystroglycan dimers. This dimeric structure was preserved in *mdx* cardiac tissue ^52^, suggesting that while the overall abundance of the dystroglycan sub-complex is decreased in dystrophic tissue, its oligomerisation status appears to be unaffected. Similarly, in this study the crosslinked dystroglycan-containing oligomeric complex of approximately 260 kDa is detected in both wild type and *mdx-4cv* skeletal muscle. Chemical crosslinking of the acetylcholine receptor-rich membrane from Torpedo electric tissue using the crosslinker succinimidyl 4-(p-maleimidophenyl) butyrate, which reacts with both free sulfhydryls and primary amines, was used to identify potential interacting partners of rapsyn, a 43 kDa receptor-associated protein of the synapse ^53^. This protein contains up to 90% of the available free sulfhydryls in acetylcholine receptor-rich membranes and so is highly amenable to crosslinking using succinimidyl 4-(p-maleimidophenyl) butyrate. This analysis identified a crosslinker-stabilised band of 90 kDa, which was immuno-labelled by antibodies to both β-dystroglycan and rapsyn. Affinity chromatography and ligand blot assays confirmed this interaction, and strongly supports the idea that dystroglycan is involved in synaptogenesis ^53^.

Overall, chemical crosslinking analysis suggests that β-dystroglycan can oligomerise with a variety of proteins to form protein complexes in cardiac and skeletal muscle. Since these protein complexes have been shown to exist in both normal and dystrophic tissue, it would appear that β-dystroglycan protein complexes can form independently of dystrophin. This has also been demonstrated by Johnson and colleagues ^47^ who could show by immuno-depletion experiments that the removal of α/β-dystroglycan results in the almost complete co-depletion of full-length dystrophin. This indicates that a considerable portion of dystrophin isoform Dp427 exists in a tight complex with α/β-dystroglycan. However, following immuno-depletion of dystrophin, a large amount of the α/β-dystroglycan sub-complex was still detectable, suggesting that not all dystroglycan molecules interact directly with dystrophin ^47^.

## Conclusions

This report has demonstrated the bioanalytical usefulness of combining chemical crosslinking, gel electrophoresis and immunoblotting for the biochemical assessment of protein oligomerisation patterns in relation to muscular dystrophy. The successful antibody-based visualization of high-molecular-mass complexes that contain the integral glycoprotein β-dystroglycan show that muscle membrane proteins of relatively low abundance can be studied by gel-shift analysis and immunoblotting. Hence, in cases where low copy number protein species involved in the molecular pathogenesis of dystrophinopathy cannot be properly identified by mass spectrometry following chemical crosslinking, simple immunochemical approaches can be employed as an alternative for the specific detection of these pathobiochemical factors.

## Data availability

The data underlying this study is available from Open Science Framework. Dataset 1: Chemical crosslinking analysis of b-dystroglycan in dystrophin-deficient skeletal muscle. DOI: http://doi.org/10.17605/OSF.IO/PNF38 ^54^

This dataset is available under a CC-By Attribution 4.0 International Licence
