# Supplementary material for: Chemical crosslinking analysis of β-dystroglycan in dystrophin-deficient skeletal muscle
Source: HRB Open Res. 2018 Sep 17;1:17. Originally published 2018 May 30. [Version 2] doi: 10.12688/hrbopenres.12846.2 (PMC9039762; doi:10.12688/hrbopenres.12846.2)
Supplement: Peer review history

# Peer review: Reviewer response for version 2

**DOI:** 10.21956/hrbopenres.13944.r26379

**Published:** 2018-09-28

**Authors:** Federica Montanaro

**Affiliations:** 1 Dubowitz Neuromuscular Centre (DNC), Molecular Neurosciences Section, Developmental Neurosciences Programme, UCL Great Ormond Street Institute of Child Health, London, UK

I like the revisions that the authors have made and do not have any further concerns. I thank them for the additional information and experiments they have made that I feel help increase the interest of this work.

Is the work clearly and accurately presented and does it cite the current literature?

Partly

If applicable, is the statistical analysis and its interpretation appropriate?

Not applicable

Are all the source data underlying the results available to ensure full reproducibility?

Partly

Is the study design appropriate and is the work technically sound?

Partly

Are the conclusions drawn adequately supported by the results?

Yes

Are sufficient details of methods and analysis provided to allow replication by others?

Yes

Reviewer Expertise:

neuromuscular disorders; gene therapy; cardiomyopathy

I confirm that I have read this submission and believe that I have an appropriate level of expertise to confirm that it is of an acceptable scientific standard.

# Peer review: Reviewer response for version 1

**DOI:** 10.21956/hrbopenres.13908.r26325

**Published:** 2018-08-01

**Authors:** Federica Montanaro

**Affiliations:** 1 Dubowitz Neuromuscular Centre (DNC), Molecular Neurosciences Section, Developmental Neurosciences Programme, UCL Great Ormond Street Institute of Child Health, London, UK

This work describes the use of chemical cross-linking to compare dystroglycan-containing complexes in wild type versus dystrophin-deficient mouse skeletal muscle. The conclusions are that 1) dystroglycan does form protein complexes in dystrophic muscle lacking full length dystrophin; and 2) chemical cross-linking in combination with standard western blotting techniques can be used to visualise oligomeric protein complexes. However, the authors do not find protein complexes that uniquely distinguish wild type from dystrophic muscle, nor do they report the protein composition of the complexes they do identify.

 In the discussion, the authors state that ‘the most interesting technical aspects of this study is the demonstration that the oligomerisation of low-abundance proteins can be evaluated by’ western blotting. From a technical standpoint, the use of cross-linkers to induce protein interactions in combination with western blotting is not novel. Cross-linking has already been applied by other groups to study the interactions of members of the dystrophin-associated protein complex. It would have been interesting if the authors had discussed here how their cross-linker differs from the ones used in prior studies and had discussed their findings in the context of a richer body of literature. In addition, cross-linkers have known challenges and can create artefacts; the authors did not include controls in their experiments to check for possible artefacts nor have they discussed them.

 The authors do note however that the cross-linked complexes containing dystroglycan are of unexpectedly low molecular weight. Indeed, beta-dystroglycan should form oligomers with the two other proteins it is known to directly interact with: dystrophin of 427kDa and alpha-dystroglycan that in muscle has an apparent molecular weight of about 150kDa. A complex of beta- and alpha-dystroglycans in association with dystrophin would be expected to generate an oligomeric complex of over 600kDa, which would have been a challenge to detect on a 6% 1D-SDS PAGE gel. The question therefore remains as to the composition of the smaller dystroglycan-containing oligomeric complexes detected here in both wild type and dystrophin-deficient muscles. An intriguing possibility is that they correspond to the novel dystroglycan complexes that our group reported in 2013 ^1^. We identified and provided a partial characterisation of non-dystrophin containing dystroglycan complexes by successfully combining immunoprecipitation with mass spectrometry in total muscle lysates from wild type and dystrophin-deficient mouse muscles. It would be interesting to test by western blotting whether the beta-dystroglycan complexes of about 260kDa reported here contain any of the new proteins we reported, namely Cavin-1 and Cavb2. In addition, the authors should test whether syntrophins and dystrobrevins are included in these complexes, possibly drawing a link to the 2003 report by the group of Mornet in Xenopus muscle that uses a similar cross-linker approach.

 The authors report using immunoprecipitation in combination with mass spectrometry to study dystroglycan-containing complexes. They encountered issues with high background and low number of peptides detected for protein identifications that they attributed to the low abundance of dystroglycan. However, from our experience, it seems more likely that either their immunoprecipitation protocol did not sufficiently enrich for dystroglycan, or the high amount of immunoglobulins in their samples interfered with MS protein identification. Have the authors checked by western blotting how much they are enriching for dystroglycan by immunoprecipitation with the two antibodies? Have they looked at how immunoglobulins are affecting their MS protein identifications? What negative controls have they included to ascertain the presence of contaminating proteins and identify them? Why have the authors not included a table with the MS results for the proteins they did identify?

 Overall, the work described here in its current form lacks information on important experimental controls, and could benefit from a few additional experiments as well as a broader citation of relevant literature to provide context and boost interest.

Is the work clearly and accurately presented and does it cite the current literature?

Partly

If applicable, is the statistical analysis and its interpretation appropriate?

Not applicable

Are all the source data underlying the results available to ensure full reproducibility?

Partly

Is the study design appropriate and is the work technically sound?

Partly

Are the conclusions drawn adequately supported by the results?

Yes

Are sufficient details of methods and analysis provided to allow replication by others?

Yes

Reviewer Expertise:

neuromuscular disorders; gene therapy; cardiomyopathy

I confirm that I have read this submission and believe that I have an appropriate level of expertise to confirm that it is of an acceptable scientific standard, however I have significant reservations, as outlined above.

## References

[1] : Identification of new dystroglycan complexes in skeletal muscle. *PLoS One* .2013;8(8) : 10.1371/journal.pone.0073224 e73224 10.1371/journal.pone.0073224 23951345PMC3738564

# Response

**Published:** 2018-09-10

**Authors:** Kay Ohlendieck

**Affiliations:** National University of Ireland, Maynooth, Ireland

The authors would like to thank Dr. Federica Montanaro for her constructive criticism and extremely helpful suggestions to improve the biochemical analysis of beta-dystroglycan oligomerisation. As suggested, we have added an additional immunoblot analysis of crosslinked cavin-1 and improved our discussion of results and related crosslinking studies, including the addition of several relevant references. Please see below our point-by-point response to major criticisms, highlighted in BOLD.

Reviewer Comment 1: This work describes the use of chemical cross-linking to compare dystroglycan-containing complexes in wild type versus dystrophin-deficient mouse skeletal muscle. The conclusions are that 1) dystroglycan does form protein complexes in dystrophic muscle lacking full length dystrophin; and 2) chemical cross-linking in combination with standard western blotting techniques can be used to visualise oligomeric protein complexes. However, the authors do not find protein complexes that uniquely distinguish wild type from dystrophic muscle, nor do they report the protein composition of the complexes they do identify.

**Authors’ response:  We would like to thank the reviewer for her detailed discussion of dystroglycan biochemistry and her constructive and critical assessment of our study. In below responses to individual comments, we have discussed in detail the changes made to the revised version of our manuscript in order to address the main points of criticism. This includes the provision of an additional immunoblot analysis, the incorporation of additional references and a discussion of our attempt to analyse beta-dystroglycan using the immuno precipitation technique. Importantly, we would like to point out that this minor study on dystroglycan oligomerisation was originally submitted to HRB Open Research as a ‘Research Note’. The category of Research Notes is defined as single-finding papers that can be reported with one or two illustrations (figures/tables), descriptions of unexpected observations, and lab protocols. However, this study has now developed into a more detailed research article. Our analysis published in HRB Open Research is an extension of a much larger and more complex investigation on the comparative and gel-based proteomic analysis of chemically crosslinked complexes in dystrophic skeletal muscle, and has recently been published (Murphy et al.: Electrophoresis. 2018; 39(14): 1735-1744). This previous investigation, which findings are now outlined in more detail in the revised Discussion section (as described in below responses), established that several proteins exhibit an increased tendency of oligomerisationin in dystrophin-deficient muscle. Mass spectrometrically identified proteins included the sarcolemmal proteins myoferlin and caveolin, the cytoskeletal components vimentin and tubulin, extracellular collagen and the mitochondrial trifunctional enzyme and oxoglutarate dehydrogenase. In agreement with the almost complete loss of full-length dystrophin in dystrophic muscle, the mass spectrometric identification of proteins with a reduced gel electrophoretic mobility following chemical crosslinking revealed the exclusive presence of dystrophin isoform Dp427 in the fraction from normal muscle. However, we were not able to identify other members of the core dystrophin‐glycoprotein complex in the dystrophin-containing gel zone that represented extremely high‐molecular-mass protein assemblies following chemical crosslinking. This is probably due to the relatively low abundance, small size and hydrophobicity of dystrophin‐associated proteins, such as beta-dystroglycan (Murphy et al.: Electrophoresis. 2018; 39(14): 1735-1744). Based on these negative findings in our previous study, we have then used immunoblotting to detect beta-dystroglycan following chemical crosslinking, and these analyses are presented here as an addition to our extensive crosslinking study recently published in Electrophoresis, and form the basis of our research study in HRB Open Research.**

**Updated Reference [24]: Murphy S, Zweyer M, Mundegar R, Swandulla D, and Ohlendieck K. Comparative gel‐based proteomic analysis of chemically crosslinked complexes in dystrophic skeletal muscle. Electrophoresis. 2018;39: 1735-44.**

Reviewer Comment 2: In the discussion, the authors state that ‘the most interesting technical aspects of this study is the demonstration that the oligomerisation of low-abundance proteins can be evaluated by’ western blotting. From a technical standpoint, the use of cross-linkers to induce protein interactions in combination with western blotting is not novel. Cross-linking has already been applied by other groups to study the interactions of members of the dystrophin-associated protein complex. It would have been interesting if the authors had discussed here how their cross-linker differs from the ones used in prior studies and had discussed their findings in the context of a richer body of literature. In addition, cross-linkers have known challenges and can create artefacts; the authors did not include controls in their experiments to check for possible artefacts nor have they discussed them.

**Authors’ response: We agree with the observation that our Research Note could benefit from a greater discussion of the literature in relation to the use of chemical cross-linking in determining interactions of members of the dystrophin-associated protein complex. Our findings are now discussed in more detail in relation to previous studies in the revised Results and Discussion sections.**

**Revised Results section: ‘…Artefacts of random crosslinking are known challenges in chemical crosslinking experiments, although it has been suggested that only those proteins that are non-randomly closely associated will become crosslinked with sufficient frequency to be detected amongst the background of all other proteins. To minimize any potential artefacts, conditions regarding buffer composition, temperature, pH-value and length of incubation time, parameters which have been previously optimised by our laboratory for muscle membrane proteins [Refs.proteins 24,31,33-35], were carefully controlled. In particular, the protein-crosslinker conjugation reaction was carried out under experimental conditions, which are relatively close to physiological conditions and low levels of crosslinker were used (10µg BS3 per mg protein)...’.**

**Revised Discussion section: ‘…In recent years a number of groups have utilised chemical crosslinking as a means of studying protein interactions in the context of the dystrophin-associated glycoprotein complex. Royuela and colleagues [Ref.51] have used the chemical N, N’-p-phenylenedimaleimide (pPDM) on enriched β-dystroglycan preparations from skeletal and cardiac muscle and brain from Xenopus laevis. Immunoblotting revealed that the 38 kDa β-dystroglycan preparation from skeletal muscle gave rise to a doublet band of 125 kDa, which was suggested to correspond to interactions between β-dystroglycan and α-dystrobrevin [Ref.51]. Although smaller in size than the crosslinked band of approximately 260 kDa established in this report, it is possible that the 260 kDa band may similarly correspond to a complex of β-dystroglycan with syntrophins and dystrobrevins, although this would need to be confirmed by immunoblotting. The pPDM agent used in the previous study [Ref.51] is based on a chemical interaction between two different cysteine groups or between one cysteine group and a lysine group. This reaction pattern differs from the BS3 XL agent used in this study, which links two primary amine groups together. The use of different cross-linking agents is also useful for determining spatial constraints given that they differ in their spacer arm length. For example, chemical crosslinkers with extremely short spacer arm lengths, such as N-succinimidyl-iodoacetate, can only stabilise the interactions between proteins that exist in very close proximity to another.**

**Our laboratory has previously used the 1,5-difluoro-2,4-dinitrobenzene crosslinker of shorter arm length than the BS3 agent used in this study to examine the cardiac dystroglycan complex [Ref.52]. A crosslinker-stabilised protein complex of approximately 200 kDa was detected, which probably represents tightly associated α/β-dystroglycan dimers. This dimeric structure was preserved in mdx cardiac tissue [Ref.52], suggesting that while the overall abundance of the dystroglycan sub-complex is decreased in dystrophic tissue, its oligomerisation status appears to be unaffected. Similarly, in this study the crosslinked dystroglycan-containing oligomeric complex of approximately 260 kDa is detected in both wild type and mdx-4cv skeletal muscle. Chemical crosslinking of the acetylcholine receptor-rich membrane from Torpedo electric tissue using the crosslinker succinimidyl 4-(p-maleimidophenyl) butyrate, which reacts with both free sulfhydryls and primary amines, was used to identify potential interacting partners of rapsyn, a 43 kDa receptor-associated protein of the synapse [Ref.53]. This protein contains up to 90% of the available free sulfhydryls in acetylcholine receptor-rich membranes and so is highly amenable to cross-linking using succinimidyl 4-(p-maleimidophenyl) butyrate. This analysis identified a crosslinker-stabilised band of 90 kDa, which was immuno-labelled by antibodies to both β-dystroglycan and rapsyn. Affinity chromatography and ligand blot assays confirmed this interaction, and strongly supports the idea that dystroglycan is involved in synaptogenesis [Ref.53].**

**Overall, chemical crosslinking analysis suggests that β-dystroglycan can oligomerise with a variety of proteins to form protein complexes in cardiac and skeletal muscle. Since these protein complexes have been shown to exist in both normal and dystrophic tissue, it would appear that β-dystroglycan protein complexes can form independently of dystrophin. This has also been demonstrated by Johnson and colleagues [Ref.47] who could show by immuno-depletion experiments that the removal of α/β-dystroglycan results in the almost complete co-depletion of full-length dystrophin. This indicates that a considerable portion of dystrophin isoform Dp427 exists in a tight complex with α/β-dystroglycan. However, following immuno-depletion of dystrophin, a large amount of the α/β-dystroglycan sub-complex was still detectable, suggesting that not all dystroglycan molecules interact directly with dystrophin [Ref.47].**

**New References:**

**[47] Johnson EK, Li B, Yoon JH, Flanigan KM, Martin PT, Ervasti J, Montanaro F. 2003. Identification of new dystroglycan complexes in skeletal muscle. PLoS One, 8(8):e73224.**

**[51] Royuela M, Chazalette D, Hugon G, Paniagua R, Guerlavais V, Fehrentz JA, Martinez J, Labbe JP, Rivier F, Mornet D. 2003. Formation of multiple complexes between beta-dystroglycan and dystrophin family products. J Muscle Res Cell Motil. 24(7): 387-97.**

**[52] Lohan J, Culligan K, and Ohlendieck K. 2005. Deficiency in Cardiac Dystrophin Affects the Abundance of the α-/β-Dystroglycan Complex. J Biomed Biotechnol. 2005(1): 28–36.**

**[53] Cartaud A, Coutant S, Petrucci TC, Cartaud J. 1998. Evidence for in situ and in vitro association between beta-dystroglycan and the subsynaptic 43K rapsyn protein. Consequence for acetylcholine receptor clustering at the synapse. J Biol Chem. 273(18):11321-6.**

Reviewer Comment 3: The authors do note however that the cross-linked complexes containing dystroglycan are of unexpectedly low molecular weight. Indeed, beta-dystroglycan should form oligomers with the two other proteins it is known to directly interact with: dystrophin of 427kDa and alpha-dystroglycan that in muscle has an apparent molecular weight of about 150kDa. A complex of beta- and alpha-dystroglycans in association with dystrophin would be expected to generate an oligomeric complex of over 600kDa, which would have been a challenge to detect on a 6% 1D-SDS PAGE gel. The question therefore remains as to the composition of the smaller dystroglycan-containing oligomeric complexes detected here in both wild type and dystrophin-deficient muscles. An intriguing possibility is that they correspond to the novel dystroglycan complexes that our group reported in 2013(Ref.1). We identified and provided a partial characterisation of non-dystrophin containing dystroglycan complexes by successfully combining immunoprecipitation with mass spectrometry in total muscle lysates from wild type and dystrophin-deficient mouse muscles. It would be interesting to test by western blotting whether the beta-dystroglycan complexes of about 260kDa reported here contain any of the new proteins we reported, namely Cavin-1 and Cavb2. In addition, the authors should test whether syntrophins and dystrobrevins are included in these complexes, possibly drawing a link to the 2003 report by the group of Mornet in Xenopus muscle that uses a similar cross-linker approach.

**Authors’ response: We would like to thank the reviewer for these comments, and have accordingly performed the suggested immunoblot analysis with antibodies to Cavin-1 and Cavb2. Unfortunately immunoblotting with Cavb2 did not result in sufficient immuno-labelling to draw any meaningful conclusions. We were not able to evaluate for the presence of syntrophins and dystrobrevins due to the lack of suitable antibodies to these dystrophin-associated proteins. The description of the successful analysis of cavin-1 has been added to the revised Abstract, Methods, Results and Discussion sections.**

**Revised Methods section: ‘Materials: … Novus Biologicals, Littleton, CO, USA (antibody NBP1-80220 to cavin 1/PTRF (polymerase and transcript release factor); RRID: AB_11019160) and …’.**

**Revised Figure 3: Comparative chemical crosslinking analysis of β-dystroglycan, cavin-1, myoferlin and dysferlin from normal versus dystrophic muscle. Shown is a silver-stained SDS-PAGE gel of muscle microsomes from wild type and dystrophic mdx-4cv skeletal muscles and corresponding immunoblots that were labelled with antibodies to β-dystroglycan, cavin-1, myoferlin and dysferlin. Lanes 1 to 4 represent microsomal samples from wild type (wt) and mdx-4cv muscles that were incubated with 0 versus 10 μg of the crosslinker bis(sulfosuccinimidyl)suberate (BS³) per mg protein, respectively. Molecular mass standards (in kDa) are indicated on the left of the gel panel. The position of monomeric versus crosslinker (XL)-stabilized oligomeric forms of β-dystroglycan, cavin-1 (CAV1) myoferlin (MYF) and dysferlin (DYF) are marked by arrowheads.**

**Revised Abstract: ‘…The membrane repair proteins dysferlin and myoferlin, which are essential components of fibre regeneration and counteract the dystrophic phenotype, as well as the caveolae-associated protein cavin-1, were also shown to exist in high-molecular mass complexes’.**

**Revised Results section: ‘Immunoblotting with antibodies to cavin-1 identified this caveolae-associated protein in both control and crosslinked samples from both wild type and mdx-4cv muscle preparations (Figure 3). In control lanes, a cavin-1 band was identified at approximately 80 kDa, which is higher than the previously observed electrophoresed protein band of the apparent monomer at 43 kDa, so this band might present a dimeric structure. The immuno-decoration at a higher molecular mass may also be due to post-translational modifications, abnormal electrophoretic mobility patterns and/or incomplete protein reduction during sample preparation for gel electrophoretic separation. In both wild type and mdx-4cv crosslinked preparations, cavin-1 was detected at lower amounts at approximately 80 kDa than in control lanes, and 3 additional bands were evident at approximately 100, 150 and 260 kDa (Figure 3). This indicates that cavin-1 may be involved in a number of protein interactions giving rise to these 3 distinct bands in crosslinked muscle samples. The fainter upper cavin-1 band of approximately 260 kDa overlaps with the position of crosslinked β-dystroglycan, which suggests that a certain proportion of cavin-1 molecules may exist in a protein complex with β-dystroglycan’.**

**Revised Discussion section: ‘…Interestingly, the sarcolemma-associated protein cavin-1 was previously shown to interact with β-dystroglycan [Ref.47]. The adapter protein cavin and the structural caveolin proteins mediate the formation and organization of caveolae. These bulb-like membrane pits represent small invaginations in the sarcolemma and form specialized lipid micro-domains. Sarcolemmal caveolae mediate a variety of processes, including cell signalling, endocytosis, lipid storage and mechano-protection [Ref.48]. The shift of both β-dystroglycan and cavin-1 to approximately 260 kDa is in agreement with the study by Johnson and co-workers [Ref.47]. However, in contrast to the previous observation that binding of cavin-1 to β-dystroglycan is lost in mdx mice suggesting that this caveolae component is reduced in the absence of dystrophin, in our study cavin-1 was detected at 260 kDa in both wild type and mdx-4cv muscle microsomes.**

**Therefore, further analysis would be required in order to determine whether the cavin-1 containing 260 kDa band also incorporates β-dystroglycan or whether this assembly represents a different cavin-1 complex’.**

**New References:**

**[47] Johnson EK, Li B, Yoon JH, Flanigan KM, Martin PT, Ervasti J, Montanaro F. 2003. Identification of new dystroglycan complexes in skeletal muscle. PLoS One, 8(8):e73224.**

**[48] Lo HP, Hall TE, Parton RG. Mechanoprotection by skeletal muscle caveolae. Bioarchitecture. 2016;6(1):22-7.**

Reviewer comment 4: The authors report using immunoprecipitation in combination with mass spectrometry to study dystroglycan-containing complexes. They encountered issues with high background and low number of peptides detected for protein identifications that they attributed to the low abundance of dystroglycan. However, from our experience, it seems more likely that either their immunoprecipitation protocol did not sufficiently enrich for dystroglycan, or the high amount of immunoglobulins in their samples interfered with MS protein identification. Have the authors checked by western blotting how much they are enriching for dystroglycan by immunoprecipitation with the two antibodies? Have they looked at how immunoglobulins are affecting their MS protein identifications? What negative controls have they included to ascertain the presence of contaminating proteins and identify them? Why have the authors not included a table with the MS results for the proteins they did identify?

**Authors’ response: We agree with the reviewer that it is possible that our immuno precipitation protocol did not sufficiently enrich for dystroglycan, or that the high levels of immunoglobulins in our samples interfered with mass spectrometry-based protein identification. We did use western blotting to check for the enrichment of β-dystroglycan in our samples pre- and post-immuno precipitation. However, while Ponceau staining of the membrane shows a band at approximately 45 kDa (possibly corresponding to β-dystroglycan), particularly when using the Abcam antibody, the detection of β-dystroglycan by western blotting appears to have been masked by the detection of immunoglobulins. Crosslinking the antibody to the magnetic beads may have helped eliminate this contamination by immunoglobulins.**

**It is important to stress that the immuno precipitation procedures described in this Research Note were initial optimisation studies to determine whether one antibody (Abcam versus Santa Cruz) or method (urea and trypsin-based elution versus elution with Laemmli-buffer followed by the FASP procedure) was superior for the enrichment of β-dystroglycan. Since none of the four experiments were deemed to be successful given that β-dystroglycan was only detected by 2 peptides, we decided not to further pursue the immuno precipitation approach. For this reason, only one replicate was performed per experiment and no control immuno precipitations using an irrelevant isotype-matched antibody were carried out. Given that these were optimisation experiments and lack a control immuno precipitation, we have chosen not to include a table with the mass spectrometry results.**

Reviewer comment 5: Overall, the work described here in its current form lacks information on important experimental controls, and could benefit from a few additional experiments as well as a broader citation of relevant literature to provide context and boost interest.

References

1. Johnson EK, Li B, Yoon JH, Flanigan KM, Martin PT, Ervasti J, Montanaro F: Identification of new dystroglycan complexes in skeletal muscle.PLoS One. 2013; 8 (8): e73224.

**Authors’ response: Our response to these comments on the lack of information on important experimental controls and the benefit of additional experiments has already been outlined above. As suggested, we have incorporated in the revised manuscript additional references [47, [48], [51, [52] and [53], as also described in detail in above responses to the reviewer’s comments:**

**[47] Johnson EK, Li B, Yoon JH, Flanigan KM, Martin PT, Ervasti J, Montanaro F. 2003. Identification of new dystroglycan complexes in skeletal muscle. PLoS One, 8(8):e73224.**

**[48] Lo HP, Hall TE, Parton RG. Mechanoprotection by skeletal muscle caveolae. Bioarchitecture. 2016;6(1):22-7.**

**[51] Royuela M, Chazalette D, Hugon G, Paniagua R, Guerlavais V, Fehrentz JA, Martinez J, Labbe JP, Rivier F, Mornet D. 2003. Formation of multiple complexes between beta-dystroglycan and dystrophin family products. J Muscle Res Cell Motil. 24(7): 387-97.**

**[52] Lohan J, Culligan K, and Ohlendieck K. 2005. Deficiency in Cardiac Dystrophin Affects the Abundance of the α-/β-Dystroglycan Complex. J Biomed Biotechnol. 2005(1): 28–36.**

**[53] Cartaud A, Coutant S, Petrucci TC, Cartaud J. 1998. Evidence for in situ and in vitro association between beta-dystroglycan and the subsynaptic 43K rapsyn protein. Consequence for acetylcholine receptor clustering at the synapse. J Biol Chem. 273(18):11321-6.**

# Peer review: Reviewer response for version 1

**DOI:** 10.21956/hrbopenres.13908.r26254

**Published:** 2018-06-13

**Authors:** John J. Mackrill

**Affiliations:** 1 Department of Physiology, BioSciences Institute, University College Cork, Cork, Ireland

The work reported in this article compares chemical cross-linking with immunoprecipitation, to characterise complexes containing the protein beta-dystroglycan in muscles from wild type and dystrophic (mdx-4cv) mice. A key finding of this work is that the reduction in the abundance of beta-dystroglycan in dystrophic mice does not alter its ability to form oligomeric complexes. In addition, immunoblotting is reported to be superior to mass-spectroscopy in terms of identifying components of cross-linked or immunoprecipitated protein complexes.

 This article is written to a very high standard and will be readily comprehended by researchers with biological or medical backgrounds. It should be of interest to researchers in the areas of muscular dystrophy, muscle biology, and to those characterising protein complexes.

 The background to this study is explained in a clear, comprehensive and concise manner. The methods are also clearly defined. The results are presented to a high standard and are discussed appropriately.

 I only have two criticisms:

1. In Figure 3, cross-linked dysferlin and myoferlin from mdx-4cv muscle display additional bands compared with those from the wild type mice. These additional bands, of higher apparent molecular weight than the monomeric forms, were not described or discussed at any point. However, this is a potentially important difference between normal and dystrophic animals; and
2. It could have been useful to apply quantitative and statistical analyses to some of these experiments, eg. in terms of quantification of protein abundances; or degree of cross-linking, etc.

Is the work clearly and accurately presented and does it cite the current literature?

Yes

If applicable, is the statistical analysis and its interpretation appropriate?

Not applicable

Are all the source data underlying the results available to ensure full reproducibility?

Yes

Is the study design appropriate and is the work technically sound?

Yes

Are the conclusions drawn adequately supported by the results?

Yes

Are sufficient details of methods and analysis provided to allow replication by others?

Yes

Reviewer Expertise:

NA

I confirm that I have read this submission and believe that I have an appropriate level of expertise to confirm that it is of an acceptable scientific standard.

# Response

**Published:** 2018-09-10

**Authors:** Kay Ohlendieck

**Affiliations:** National University of Ireland, Maynooth, Ireland

The authors would like to thank Dr. John Mackrill for his encouraging and positive remarks on our study and his helpful comments to improve the interpretation of the key findings from our chemical crosslinking analysis of beta-dystroglycan. Please see below our point-by-point response to his two major criticisms, highlighted in BOLD.

Reviewer comment 1: I only have two criticisms: (1) In Figure 3, cross-linked dysferlin and myoferlin from mdx-4cv muscle display additional bands compared with those from the wild type mice. These additional bands, of higher apparent molecular weight than the monomeric forms, were not described or discussed at any point. However, this is a potentially important difference between normal and dystrophic animals.

**Authors’ response: We agree with the reviewer that this is an interesting observation and have added this point to the revised Results and Discussion sections:**

**Revised Results section; ‘… This limited reduction in electrophoretic mobility of myoferlin and dysferlin in both wild type and dystrophic skeletal muscle, upon the addition of the BS3 chemical cross-linker, suggests that only a sub-population of these proteins form tight protein complexes. The presence of additional bands representing these proteins in mdx-4cv muscle indicates that in dystrophic muscle these proteins probably form additional protein complexes in a dynamic fashion, and that some of these protein assemblies are not present in wild type skeletal muscle’.**

**Revised Discussion section: ‘… The more diverse protein complex formation of dysferlin and myoferlin in mdx-4cv membranes, as compared to wild type muscle, may be a compensatory mechanism of dystrophic skeletal muscle to mitigate some of the secondary pathobiochemical effects, which arise as a result of the loss of dystrophin. Indeed, our research group has recently used chemical crosslinking combined with mass spectrometry to investigate alterations in the oligomerisation of high** **‐** **molecular** **‐** **mass protein complexes in dystrophic skeletal muscle [Ref.24]. This analysis identified 23 proteins in dystrophic microsomal fractions, which displayed an electrophoretic mobility shift to very high molecular mass, of which 15 proteins were only found in dystrophic microsomes and 8 were detected in both wild type and mdx-4cv microsomes. One of these 15 proteins was identified as myoferlin [Ref.24], which may interact with caveolin, actin and myosin-9 in high-molecular-mass complexes, and facilitate sarcolemmal membrane repair. Since membrane repair is a well-described compensatory mechanism in dystrophic muscle, which is partially mediated by myoferlin and dysferlin, heightened interactions of these proteins with other repair proteins in muscular dystrophy may account for the additional protein complexes seen in dystrophic muscle.**

Reviewer comment 2: (2) It could have been useful to apply quantitative and statistical analyses to some of these experiments, eg. in terms of quantification of protein abundances; or degree of cross-linking, etc.

**Authors’ response:  To address this issue, we have included an additional graph corresponding to the gel image in Figure 3 which illustrates increases in densitometry values corresponding to increased protein staining in high-molecular mass regions of the gel (>150 kDa) for wild type and dystrophic cross-linked samples. Chemical crosslinking resulted in protein complex formation and reduced electrophoretic mobility of protein species, thus resulting in an increased number of proteins remaining at high molecular masses, evident here by increased densitometry values.**

**Revised Methods section: Densitometric scanning and statistical analysis of gel images was performed using a HP PSC-2355 scanner and ImageJ software (NIH, Bethesda, MD, USA) along with Graph-Pad Prism software (San Diego, CA, USA), in which statistical significance was based on a p value ≤ 0.05.**

**Revised Results section: ‘… that contain β-dystroglycan (Figure 3). The densitometric analysis of the silver-stained gel illustrates statistically significant increases in densitometry values corresponding to increased protein staining in high-molecular mass regions of the gel above 150 kDa for both wild type and dystrophic crosslinked samples (Figure 4). Hence, chemical crosslinking resulted in the stabilisation of protein complexes and reduced electrophoretic mobility of protein species, resulting in an increased number of proteins remaining at high molecular mass regions following electrophoretic separation. Interestingly the …’.**

New Figure 4: Densitometric scanning of chemical crosslinking and gel-shift analysis of normal versus dystrophic muscle. Shown are densitometric values, which reflect the extent of protein staining in wild type control, wild type crosslinked, mdx-4cv control and mdx-4cv crosslinked samples. The comparative densitometric evaluation was focused on protein species above 150 kDa. Statistical analysis was performed with a Student’s t-test (mean value ±SEM; n=4; **p≤0.01; ***p≤0.001).
